# Isolation of a novel species of flavivirus and a new strain of Culex flavivirus (*Flaviviridae*) from a natural mosquito population in Uganda

**DOI:** 10.1099/vir.0.014183-0

**Published:** 2009-11

**Authors:** Shelley Cook, Gregory Moureau, Ralph E. Harbach, Louis Mukwaya, Kim Goodger, Fred Ssenfuka, Ernest Gould, Edward C. Holmes, Xavier de Lamballerie

**Affiliations:** 1 Natural History Museum, Cromwell Road, London SW7 5BD, UK; 2 Unité des Virus Emergents UMR190 ‘Emergence des Pathologies Virales’, Université de la Méditerranée et Institut de Recherche pour le Développement, Marseille, France; 3 Mosquito Research Programme, Uganda Virus Research Institute, PO Box 49, Entebbe, Uganda; 4 Centre for Ecology and Hydrology Oxford, Mansfield Road, Oxford OX1 3SR, UK; 5 Department of Biology, The Pennsylvania State University, University Park, PA 16802, USA

## Abstract

The genus *Flavivirus*, which contains approximately 70 single-stranded, positive-sense RNA viruses, represents a unique model for studying the evolution of vector-borne disease, as it includes viruses that are mosquito-borne, tick-borne or have no known vector. Both theoretical work and field studies suggest the existence of a large number of undiscovered flaviviruses. Recently, the first isolation of cell fusing agent virus (CFAV) was reported from a natural mosquito population in Puerto Rico, and sequences related to CFAV have been discovered in mosquitoes from Thailand. CFAV had previously been isolated from a mosquito cell line in 1975 and represented the only known ‘insect-only’ flavivirus, appearing to replicate in insect cells alone. A second member of the ‘insect-only’ group, Kamiti River virus (KRV), was isolated from Kenyan mosquitoes in 2003. A third tentative member of the ‘insect-only’ group, Culex flavivirus (CxFV), was first isolated in 2007 from Japan and further strains have subsequently been reported from the Americas. We report the discovery, isolation and characterization of two novel ‘insect-only’ flaviviruses from Entebbe, Uganda: a novel lineage tentatively designated Nakiwogo virus (NAKV) and a new strain of CxFV. The individual mosquitoes from which these strains were isolated, identified retrospectively by using a reference molecular phylogeny generated using voucher specimens from the region, were *Mansonia africana nigerrima* and *Culex quinquefasciatus*, respectively. This represents the first isolation, to our knowledge, of a novel insect-only flavivirus from a *Mansonia* species and the first isolation of a strain of CxFV from Africa.

## INTRODUCTION

The genus *Flavivirus* represents a unique model for studying the evolution of vector-borne disease because it includes viruses that are: (i) arthropod-borne, infecting a range of vertebrate hosts through mosquito or tick bites; (ii) presumed to be limited to vertebrates alone; and (iii) appear to be restricted to insects alone (‘insect-only’ flaviviruses). In addition, the genus includes pathogens of significant importance to human health, including dengue virus and yellow fever virus (YFV). Cell fusing agent virus (CFAV), first isolated from a *Stegomyia aegypti* (as *Aedes aegypti*) cell line [Stollar & Thomas, 1975; nomenclature for the aedine mosquitoes follows Reinert (2000) and Reinert *et al.* (2004, 2006, 2008)] and characterized by Cammisa-Parks *et al.* (1992), previously represented the only known ‘insect-only’ flavivirus, appearing to replicate uniquely in insect cells (Kuno, 2007). In recent years, Cook *et al.* (2006) reported the first isolation of CFAV, from a natural mosquito population in Puerto Rico. The virus was found in several different species and in mosquitoes of both sexes. Two strains of a second member of the ‘insect-only’ group, designated Kamiti River virus (KRV), were isolated from *Neomelaniconion mcintoshi* mosquitoes from Kenya {Crabtree *et al.*, 2003; Sang *et al.*, 2003; these authors stated that KRV was isolated from ‘*Ae. macintoshi*’ from flooded dambos in Central Province, Kenya. However, *Aedes* (*Ochlerotatus*) *macintoshi* (Marks) [*Ochlerotatus macintoshi* in the classification of Reinert *et al.* (2008)] is an Australian mosquito, and the species in this case is actually the Rift Valley fever vector *Neomelaniconion mcintoshi* (Huang) (originally named *Aedes mcintoshi*), whose larvae are frequently found in dambos}. Taken together with theoretical work that suggested the existence of over 2000 unknown mosquito-borne flaviviruses (Pybus *et al.*, 2002), as well as the discovery of DNA sequences related to flaviviruses in the genomes of *St. aegypti* and *Stegomyia albopicta*, which probably resulted from integration events following infection of each mosquito species by a virus (or viruses) related to the CFAV group, it was proposed that further ‘insect-only’ flaviviruses were likely to be discovered in natural mosquito populations (Crochu *et al.*, 2004). Indeed, sequences related to CFAV been discovered have more recently in *St. aegypti* mosquitoes from Thailand (Kihara *et al.*, 2007). A third member of the ‘insect-only’ group, Culex flavivirus (CxFV), was first isolated in 2007 from *Culex tritaeniorhynchus* and *Culex quinquefasciatus* in Japan and Indonesia (Hoshino *et al.*, 2007). Further strains have subsequently been isolated from *Cx. quinquefasciatus* in Guatemala (Morales-Betoulle *et al.*, 2008) and Mexico (Farfan-Ale *et al.*, 2009), and from *Cx. quinquefasciatus* and *Culex restuans* in Texas, USA, and Trinidad (Kim *et al.*, 2009). Finally, flavivirus RNA has also recently been discovered in phlebotomine sandflies from Algeria (Moureau *et al.*, 2009).

We now report the discovery, isolation and characterization of two novel flaviviruses of the ‘insect-only’ group from the Entebbe area of Uganda: a novel lineage, tentatively designated Nakiwogo virus (NAKV), and a new strain of CxFV. The individual mosquitoes from which these strains were isolated, identified retrospectively from a backbone phylogeny generated using the ‘barcode’ region of voucher specimens from the area, were specimens of *Mansonia africana nigerrima* and *Cx. quinquefasciatus*, respectively. To date, insect-only flaviviruses have been isolated from *Culex*, *Stegomyia* and *Ochlerotatus* mosquitoes, in common with the majority of the mosquito-borne flaviviruses. This work represents, to our knowledge, the first isolation of a novel insect-only flavivirus from a *Mansonia* species and the first isolation of a strain of CxFV from Africa. In addition, we include in our analyses, for the first time, the full genome sequence from CFAV Rio Piedras, isolated previously from Puerto Rico (Cook *et al.*, 2006).

## METHODS

### Trapping protocol.

**Fig. 1. f1:**
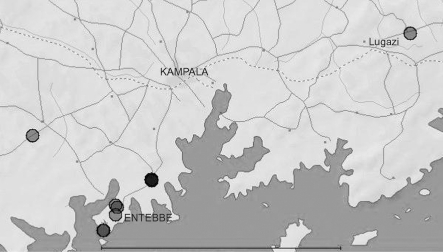
Mosquito collection sites in Uganda. Bar, 50 km.

In total, 419 individual mosquitoes were collected in February 2008 for virus screening. In addition, 204 mosquitoes representing species found at sampling locations were retained as voucher specimens for the development of molecular identification protocols. Mosquitoes were sampled from a variety of locations (Fig. 1), using different methods to maximize species diversity. Centers for Disease Control (CDC) fan-augmented light traps were supplemented with dry ice for approximately 8 h trapping periods between dusk and dawn and placed at various heights in urban, rural and forest environments. Modified backpack aspirators and hand-held aspirators were used to sample resting mosquitoes. Specimens were placed on dry ice immediately upon collection at the sampling sites. Upon arrival at the laboratory, samples were sorted on a chill table according to trap, location and sex and placed in individual wells in microtitre plates before storage at −80 °C.

### Sample preparation, nucleic acid extraction and RT-PCR screening.

Whole mosquitoes were homogenized individually as described previously (Cook *et al.*, 2006). Samples of 65 μl from five individual mosquitoes were pooled for RNA extraction. Total nucleic acid (NA) extraction was conducted via the MagNApure LC System using a Culture Cells kit and standard manufacturer's protocol (Roche Diagnostics), or using a Biorobot EZ1, with Virus Mini kit v2.0 (Qiagen).

A 5 μl aliquot of each pooled RNA extraction was used in a one-step real-time QuantiTect SYBR-Green RT-PCR assay, according to Moureau *et al.* (2007), with primers PF1S and PF2R-bis (see Supplementary Table S1, available in JGV Online). Second-round PCR on 2.5 μl cDNA, using primers PF3S and PF2R-bis, used the following conditions: 94 °C for 2 min; 40 cycles of 94 °C for 30 s, 50 °C for 45 s and 72 °C for 1 min; followed by a final extension of 72 °C for 7 min and cooling to 20 °C for 2 min. To avoid any potential contamination, positive cDNA samples from first-round RT-PCR were manipulated separately from all other samples for second-round PCR. Negative controls comprising both NA extractions from non-infected mosquitoes and reactions in which water replaced RNA were performed through all stages. Positive controls comprised Rio Bravo, Japanese encephalitis and Montana myotis leukoencephalitis viruses (RBV, JEV and MMLV, respectively).

Second-round PCR products were analysed visually by electrophoresis through ethidium bromide-stained 2 % agarose gels under UV light. Products were purified by using a QIAquick Spin PCR Purification kit (Qiagen). Amplicons were sequenced on both strands with an ABI 377 automated sequencer (Applied Biosystems).

For those samples that gave positive results using the primer set PF1S–2Rbis–3S on RNA extractions from pooled mosquitoes, NA were re-extracted from the original individual mosquito homogenates by using a Biorobot EZ1, with Virus Mini kit v2.0 according to the manufacturer's instructions (Qiagen). One-step SYBR green first-round RT-PCR was followed by second-round PCR using the PF1–2bis–3 system as described for the pooled samples. Second-round PCR products were analysed visually, purified and sequenced as described above.

### Further investigation of individual samples and virus isolation.

Individual mosquito homogenates were investigated further based on (i) congruent positive sequences in both pooled sample and individual mosquitos and (ii) the resulting 157 nt NS5 sequence clustering with other known ‘insect-only’ flaviviruses.

For the individual mosquito homogenates chosen for virus isolation work, 100 μl homogenate was centrifuged at 13 000 r.p.m. (16 100 ***g***) for 20 min. Subsequently, the supernatant was diluted in a 1 ml final volume of L15 medium without fetal bovine serum (FBS), but enriched with antibiotics (ml^−1^: 100 IU penicillin G, 100 mg streptomycin, 100 mg kanamycin, 2.5 μg amphotericin B) and inoculated onto C6/36 cells in single 12.5 cm^2^ flasks. After incubation at room temperature for 1 h, 4 ml fresh 3 % FBS L15 medium was added. The flask was incubated at 28 °C. A negative-control flask was handled under identical conditions. Flasks were examined daily for the presence of cytopathic effect [i.e. cell fusion and/or syncytium formation similar to that seen in the prototype CFAV, as described by Stollar & Thomas (1975)] and 400 μl of each supernatant medium was extracted and tested by real-time RT-PCR. A series of four passages on C6/36 cells was carried out for each positive isolate.

The strategy for sequencing of the entire open reading frame (ORF) followed Grard *et al.* (2007). Initial RT-PCR amplifications targeted regions encoding the E, NS3 and NS5 proteins using degenerate primers (Crochu *et al.*, 2004; Gaunt & Gould, 2005; Gaunt *et al.*, 2001; Moureau *et al.*, 2007). Specific primers were then designed to complete gaps via long-range RT-PCR (cMaster RT_plus_PCR system; Eppendorf) and amplicons were sequenced using the long PCR product sequencing (LoPPS) method, a shotgun-based approach applied to long PCR amplification products (Emonet *et al.*, 2006, 2007). Primer sequences are available from the corresponding author upon request.

### Transmission electron microscopy.

Tissue-culture samples showing cytopathic effect were prepared for electron-microscopic examination. Negative-stained electron-microscopic specimens were prepared by drying culture supernatant medium, mixed 1 : 1 with 2.5 % paraformaldehyde, onto Formvar/carbon-coated grids and staining with 2 % methylamine tungstate.

### Investigation for the presence of virus-specific DNA.

It has been previously shown that DNA forms of CFAV are produced during infection of a number of different mosquito cell types (Cook *et al.*, 2006). Therefore, for flaviviral isolates, total NA were extracted from C6/36 cultures of each virus at passage 5, day 6 post-infection by using a Biorobot EZ-1. Both supernatant medium and sedimented cells were tested using the primer pairs detailed in Supplementary Table S1. First, sedimented cells and supernatant medium were tested by using standard PCR with no RT step. Second, the same primer pairs were used for a one-step RT-PCR (Promega). Third, NA from sedimented cells were treated with recombinant DNase I according to the manufacturer's instructions (Roche). These DNase-treated samples were then tested in a classic PCR with no RT step.

### Phylogenetic analyses of viral sequences.

Sequencher v4.8 (Gene Codes) was used to combine reverse and forward viral sequences. Sequences were then compared with those of all other members of the genus *Flavivirus* available to date. Supplementary Table S2 (available in JGV Online) lists the flaviviral sequences that were used in analyses. Datasets were prepared by using Se-Al (available at http://tree.bio.ed.ac.uk/software/seal/) for (i) nucleotide data for the region encoding the NS5 protein, (ii) amino acid alignment of the complete ORF and (iii) nucleotide and amino acid sequences for the region encoding the E protein for the insect-only flaviviruses, as this currently comprises the greatest number of species available for this group. All nucleotide analyses were repeated both including and excluding third-codon positions (as these were likely to experience site saturation).

For the NS5 region, the dataset was primarily limited to those flaviviruses for which ORF data were also available, to allow comparison between phylogenies. Nucleotides were aligned by using muscle (Edgar, 2004) with manual adjustment to maintain correct reading frame. The model of nucleotide substitution and parameter values were selected via modeltest (Posada & Crandall, 1998) and used to estimate maximum-likelihood (ML) phylogenetic trees in paup (Swofford, 2000). Values for the substitution matrix, base composition, gamma distribution of among-site rate variation (Γ) and the proportion of invariant sites (I) are available from the authors on request. A bootstrap-resampling analysis was conducted using 1000 replicate neighbour-joining (NJ) trees based on the ML substitution matrix. Phylogenetic analyses were also performed using the Bayesian method available in MrBayes v3.1.2 (Huelsenbeck & Ronquist, 2001) with a minimum of 10 million generations and a burn-in of 10 %. Stationarity was assessed at effective sample size >400 using Tracer v1.4.1 (part of the beast package; Drummond & Rambaut, 2007). For the full ORF dataset, amino acids were aligned by using muscle and phylogenetic analyses were performed in MrBayes, with a minimum of 10 million generations and a burn-in of 10 %. For the E region, nucleotides were aligned based on alignment of amino acid sequences in muscle. ML phylogenetic analyses conducted by using modeltest and paup were identical to those carried out for the NS5 region. In addition, Bayesian analyses were conducted on the amino acid dataset in MrBayes as described above, after removing 17 sites of ambiguous alignment using GBlocks (Talavera & Castresana, 2007). Final datasets comprised 60 sequences (2514 sites) for the NS5 region (corresponding to nucleotide positions 7790–10270 of YFV; GenBank accession no. X03700), 58 sequences (3801sites) for the ORF and 27 sequences (417 sites) for the E region (corresponding to amino acid positions 1184–1531 of YFV; GenBank accession no. U17067).

### Extraction, amplification and sequencing of voucher mosquito DNA.

Voucher mosquito specimens had previously been identified using morphology (Edwards, 1941; Service, 1990). A 130 μl volume of Chelex 100 Resin 15 % (w/v) (Bio-Rad) was added to 20 μl mosquito homogenate. This was incubated at 95 °C for 20 min and centrifuged at 13 000 r.p.m. (16 100 ***g***) for 15 min. Further analysis was conducted using 50–60 μl supernatant and, of this extraction, 1.0–2.5 μl was used in PCRs. DNA was also extracted, amplified and sequenced from those individual mosquito homogenates found to be positive for novel flavivirus strains.

The COI gene was amplified by using primers UEA3 (5′-TATRGCWTTYCCWCGAATAAATAA-3′) (Lunt *et al.*, 1996) and Fly10 (5′-ASTGCACTAATCTGCCATATTAG-3′) (Sallum *et al.*, 2002) according to Cook *et al.* (2006) (hereafter referred to as the ‘Fly’ region). An additional overlapping region, the ‘barcode’ section of the COI gene, was amplified using the primers LCO1490 (5′-GGTCAACAAATCATAAAGATATTGG-3′) and HCO2198 (5′-TAAACTTCAGGGTGACCAAAAAATCA-3′) [both from Folmer *et al.* (1994)] with a primer concentration of 10 μM and reaction conditions of 5 min at 95 °C; 40 cycles of 30 s at 95 °C, 30 s at 48 °C and 45 s at 72 °C; followed by a final extension time of 5 min at 72 °C. COI sequences obtained during this work have been deposited in GenBank with accession numbers GQ165759–GQ165807.

### Phylogenetic analysis of mosquito sequences.

Sequencher v4.8 was used to combine reverse and forward sequences from each mosquito and final datasets were compiled using Se-Al. These sequences were added to those currently available from GenBank and the Barcode of Life Datasystem (BOLD) (Ratnasingham & Hebert, 2007). For all voucher specimens, COI sequences from a minimum of five field-collected individuals per species were included in analyses where possible. In the case of sequences from public databases, five sequences per species (preferably from different geographical locations) were again included where available. Sequences from flavivirus-positive mosquito samples were also included. Datasets for each region comprised 1152 bp from 112 individuals and 636 bp from 338 individuals for the ‘Fly’ and ‘barcode’ regions, respectively. Analyses were conducted separately for each region, due to the fact that taxonomic coverage of mosquito species in public databases varies for each. Following confirmation that all species groups of five were monophyletic, analyses were then repeated using single representatives per species for ease of presentation, except in the case of clades including flavivirus-positive mosquitoes, in which case sequences from multiple vouchers or public data for that species were included if possible. These ‘single representative per species’ datasets comprised 138 and 46 individuals, respectively, for the ‘barcode’ and ‘Fly’ regions.

In each case, nucleotide sequences were aligned by using muscle (Edgar, 2004) with manual adjustment to maintain correct reading frame, the model of nucleotide substitution and parameter values were selected via modeltest (Posada & Crandall, 1998) and used to estimate ML phylogenetic trees in paup (Swofford, 2000). Bootstrap-resampling analyses were conducted by using 1000 replicate NJ trees based on the ML substitution matrix. For sample H3E6 in the barcode region, the ML phylogeny of *Culex* and *Culiseta* sequences only was also estimated for ease of presentation, and is shown in Fig. 6 (see Supplementary Table S3, available in JGV Online). Trees were rooted by using *Chagasia* (Fly region) or *Culiseta* (barcode subset).

## RESULTS

### Flavivirus screening

Positive and negative controls carried through all protocols showed that the system was both sensitive to the presence of flaviviruses and free from contamination. From 419 mosquitoes assayed using semi-nested RT-PCR with the PF1S–2R–3S system, four pools were PCR-positive, with the resultant sequences clustering with known ‘insect-only’ flaviviruses.

Upon further testing of the 20 individual samples that contributed to the four positive pools, three individual mosquito homogenates gave congruent positive sequences. No congruent sequences were amplified from individual mosquitoes contributing to the fourth positive pool and virus isolation from both the pool and the relevant homogenates was unsuccessful. Of the remaining three potential flavivirus strains, two individual sequences were identical and originated from mosquitoes in the same trap. Isolation was attempted using only one of these two homogenates. Therefore, in total, two flavivirus strains were taken forward for further characterization and isolation work. Nevertheless, retrospective identification was conducted for all three individual mosquitoes for which flavivirus-positive results were obtained via RT-PCR.

### Virus isolation and characterization

The first novel strain (H4A1) was isolated from a female mosquito, UG134-26, collected in a CDC light trap supplemented with dry ice on 24 February 2008 near Entebbe (latitude 0.07555867, longitude 32.4574212; Fig. 1). The second strain (H3E6) was isolated from a female mosquito, UG134-14, from the same trap. Notably, the third virus-positive sample, H4D1, which was identical at the nucleotide level to H4A1 for the PF3S/PF2R-bis amplicon, was isolated from another female mosquito, UG134-26, also captured in the same trap.

**Fig. 2. f2:**
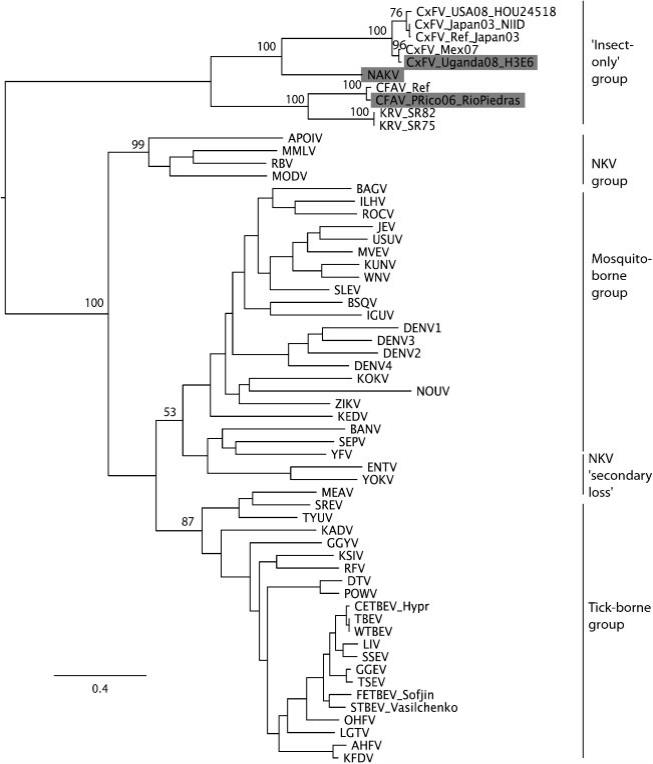
Maximum-likelihood (ML) phylogenetic tree of the novel CxFV Uganda and NAKV strains and other members of the genus *Flavivirus* for the NS5 nucleotide dataset. Bootstrap values are shown for the main clades only. See Supplementary Table S2 for virus abbreviations and GenBank accession numbers. Identical topologies were obtained using both paup (including and excluding third-codon positions) and MrBayes for the insect-only flaviviruses. All horizontal branch lengths are drawn to scale; bar, 0.4 substitutions per site. The tree is midpoint-rooted for purposes of clarity only. Those flavivirus sequences that are published for the first time in the current study, namely NAKV, CxFV strain Uganda and CFAV strain Rio Piedras, are highlighted in grey.

**Fig. 4. f4:**
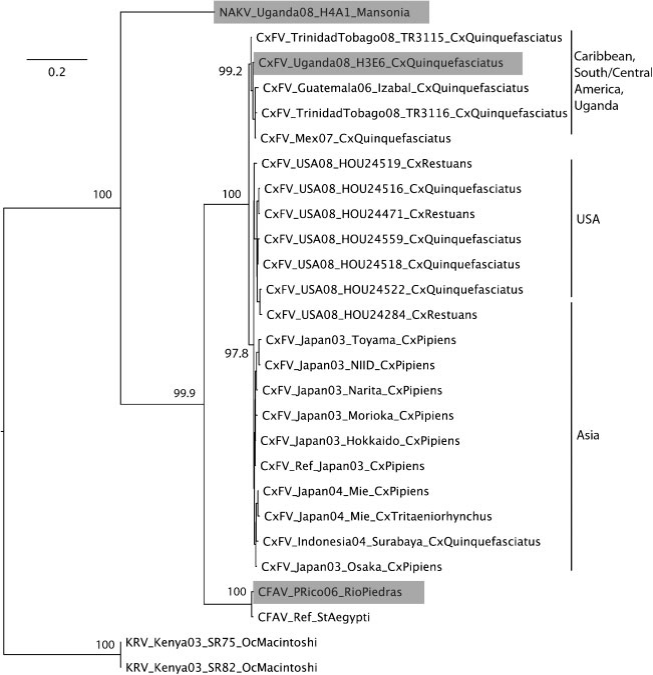
Bayesian phylogeny of the E region insect-only amino acid dataset from the genus *Flavivirus*, including CxFV and NAKV. Country and year of isolation, strain and mosquito species are shown for each virus. Posterior probabilities (%) are shown for the main clades only. See Supplementary Table S2 for virus abbreviations and GenBank accession numbers. The topology of the E region nucleotide phylogeny, both including and excluding third-codon positions, was identical to that obtained by using amino acid data. All horizontal branch lengths are drawn to scale; bar, 0.2 substitutions per site. The tree is rooted by using KRV for the purpose of clarity only (midpoint rooting produces a similar topology). Those flavivirus sequences that are published for the first time in the current study, namely NAKV, CxFV strain Uganda and CFAV strain Rio Piedras, are highlighted in grey.

The ORFs of isolate H3E6, designated CxFV Uganda, and isolate H4A1, tentatively designated Nakiwogo virus (NAKV, named after the location of the trap), are 10 092 and 10 122 nt long, respectively (GenBank accession numbers GQ165808 and GQ165809). The topology of the insect-only group, as well as its position relative to the other members of the genus, was similar in both the NS5 and ORF phylogenies (Figs 2 and 3, respectively). CxFV Uganda appears to be related most closely to CxFV from Mexico, with an uncorrected p-distance of 9.7 % in NS5. NAKV appears to form a sister group to the CxFV group, with an uncorrected p-distance between NAKV and CxFV Uganda of 40.5 %. In contrast, in the E region phylogeny shown in Fig. 4, CFAV is related more closely to CxFV than is NAKV.

**Fig. 5. f5:**
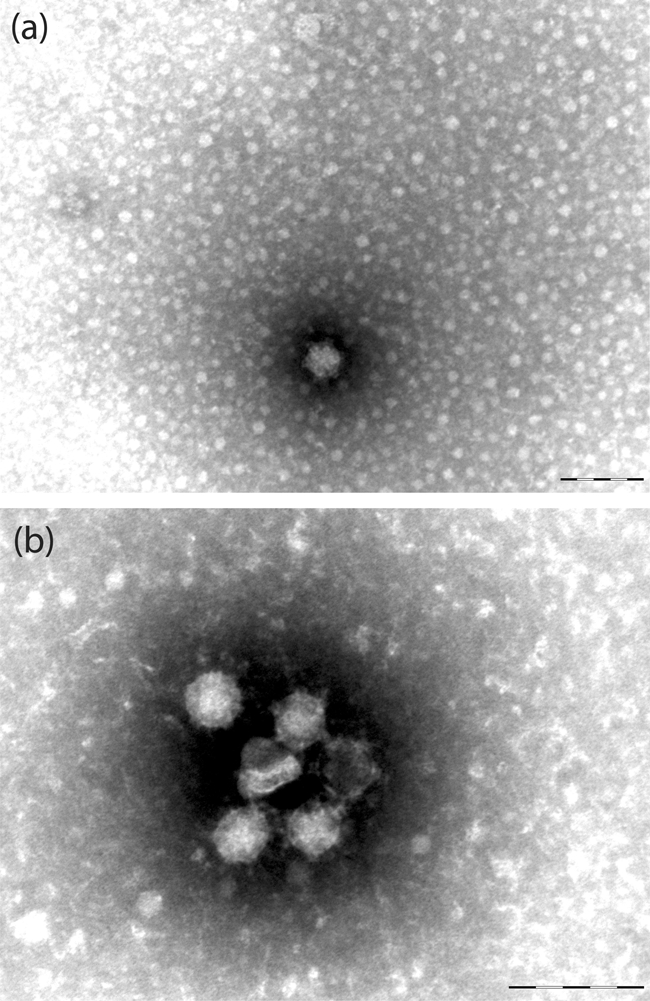
Electron micrographs of C6/36 cells infected with (a) CxFV Uganda and (b) NAKV. Bars, 100 nm.

As shown in Fig. 5, flavivirus-like particles were visible in infected C6/36 culture for both CxFV Uganda and NAKV. In common with other members of the genus, the spherical virions are enveloped with clear projections and approximately 40 nm in diameter. Cytopathic effect in C6/36 cells infected with NAKV was moderate and similar to that seen in the prototype CFAV (Stollar & Thomas, 1975), with the formation of large syncytia. In contrast, CxFV Uganda was associated with a reduction in cell density and modification of cell shape from circular to triangular.

Results from the investigation of DNA forms are shown in Table 1. For both H4A1 and H3E6, results were consistent with RNA forms of each flavivirus being present in both culture supernatant and pelleted cells. DNA forms were also present, but in sedimented cells only.

### Phylogeny and identification of flavivirus-positive mosquitoes

**Fig. 6. f6:**
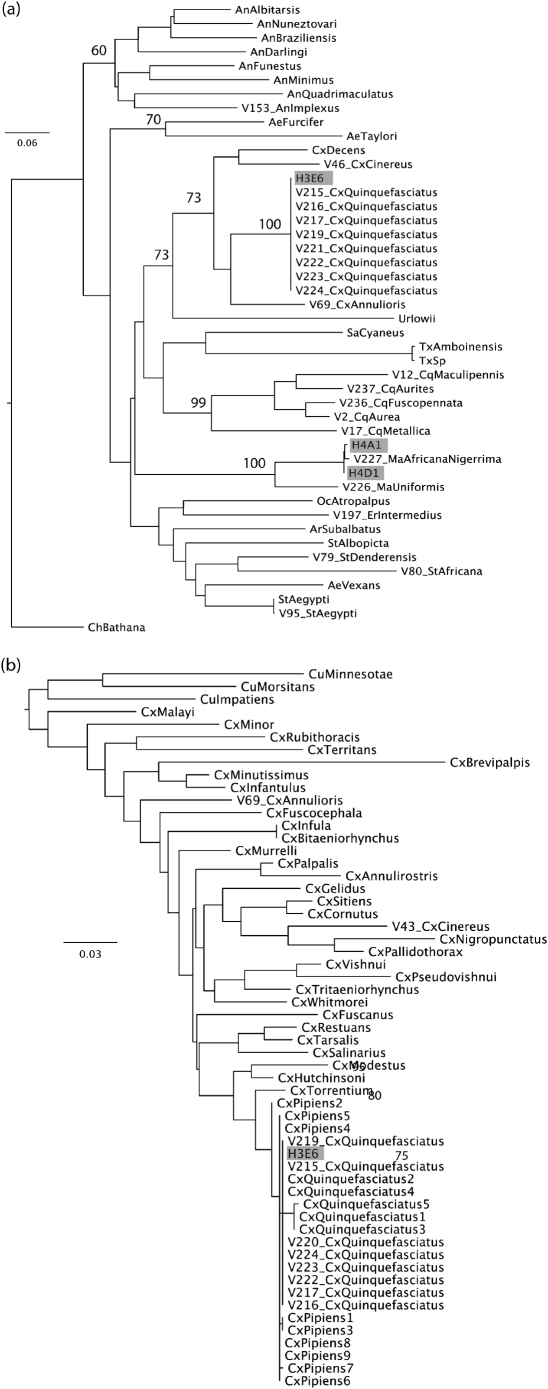
ML phylogenetic tree of mosquitoes plus virus-positive samples from Uganda for the COI gene. Bootstrap values for main clades of >50 % only are shown for clarity. All horizontal branch lengths are drawn to scale; bars, 0.06 (a) or 0.03 (b) substitutions per site. V, Field-collected voucher specimen; H3E6, mosquito from which CxFV Uganda was isolated; H4A1 and H4D1, mosquitoes from which NAKV was isolated. All other sequences are from public databases as detailed in Supplementary Table S3. (a) ‘Fly’ region of COI, rooted using *Chagasia*. (b) ‘Barcode’ region of COI, *Culex* subset, rooted using *Culiseta*.

Alignment of COI sequences from voucher and flavivirus-positive mosquitoes was unambiguous. In all cases, groups of five individuals from a given species from both the field and public databases were monophyletic, indicating, in common with other studies, that this gene is suitable for species identification (Hebert *et al.*, 2003). Further analyses, using single representatives from each species except in the case of species related to the flavivirus-positive mosquitoes (in which case, multiple representatives are included where possible), are shown in Fig. 6. Sample H3E6, from which CxFV Uganda was isolated, grouped with sequences from field-collected *Cx. quinquefasciatus* voucher specimens in both phylogenies. Mosquitoes comprising samples H4A1 and H4D1 grouped with a field-collected voucher specimen of *Ma. africana nigerrima* for the ‘Fly’ region. Primers LCO1490 and HCO2198 did not amplify the barcode region for the mosquitoes that comprised samples H4A1 and H4D1 and, for ease of presentation, we show the *Culex* subset phylogeny, including sample H3E6, for this region of the COI gene.

## DISCUSSION

We have described the first isolation, to our knowledge, of a novel flavivirus, NAKV, from a field-collected *Mansonia* mosquito. We have also isolated a novel strain of the flavivirus CxFV, designated here CxFV Uganda, from adult *Cx. quinquefasciatus* mosquitoes. Kuno *et al.* (1998) suggested that pairwise sequence identity of >84 % correlates well with among-species antigenic characteristics for the flaviviruses. As such, and considering the >30 % sequence divergence of NAKV from all other members of the group, it is possible that NAKV comprises a novel species within the genus, although all such definitions are essentially arbitrary. The approximately 10 % difference between the reference CxFV (‘Japan03’) and the Ugandan strain is comparable to that seen between other strains of CxFV.

Both the NS5 and ORF ML analyses indicate that CxFV Uganda is related most closely to CxFV from Mexico, with strong support for this clade. The larger E region dataset suggests that CxFV isolates from the Caribbean and Guatemala also fall into this group. A second CxFV lineage contains isolates from Japan and North America, which fall as sister groups. Previous studies had also identified these two main clades, namely Trinidad/Guatemala and Asia/Texas, as potential subtypes or genotypes of CxFV (Kim *et al.*, 2009). The addition of CxFV Uganda from Africa, which falls into the first of these groups, supports the hypothesis that CxFV may have been introduced multiple times into the Americas, (i) from Africa to the Caribbean, potentially via routes similar to the introduction of other mosquito-borne flaviviruses such as YFV (Gould *et al.*, 2003) and (ii) from Asia to North America (Lounibos, 2002). As such, the distribution of CxFV may reflect that of the *Culex pipiens* complex, which is cosmopolitan and ubiquitous, with multiple introductions of this mosquito species into the New World. Notably, although members of the *Cx. pipiens* complex differ in physiology, ecology and behaviour, sibling species are very difficult to distinguish from one another via morphological methods. Until molecular protocols for the accurate identification of *Culex* species across the range of the complex are fully developed, there is the potential for misidentification of the vectors of CxFV and, as such, it is difficult to draw conclusions regarding the relative prevalence and distribution of CxFV in *Cx. pipiens* and *Cx. quinquefasciatus*. As shown in Fig. 6, although the *Cx. quinquefasciatus* clade is monophyletic and the molecular identification of the mosquito species from which CxFV Uganda was isolated is robust, resolution of the *Cx. pipiens* plus *Cx. quinquefasciatus* clade is not high and the development of a molecular backbone phylogeny for these species in Uganda using an additional gene, for example ITS2 (Severini *et al.*, 1996), is recommended for future work.

NAKV tentatively forms a novel species of flavivirus that appears to be related most closely to the CxFV group in the NS5 and ORF phylogenies. CFAV and KRV then form a sister group to the NAKV/CxFV lineage. In contrast, in the E region phylogeny, CFAV is related more closely to CxFV than NAKV. Although this is compatible with potential interspecies recombination, interpretation of the E region tree is not unambiguous, in particular the position of the root, because the high genetic distances involved mean that other members of the genus *Flavivirus* were not incorporated into this analysis. It is therefore clear that additional data, particularly comprising ORFs for all available insect-only flaviviruses, would be required in future to test this more fully via formal tests.

Notably, both CxFV Uganda and the two samples of NAKV (i.e. homogenates H4A1 and H4D1, which were both positive via RT-PCR, with identical sequences) were isolated from the same trap, but from different mosquito species, namely *Cx. quinquefasciatus* and *Ma. africana nigerrima*, respectively. Both of these mosquito species were trapped in other locations and are present across Uganda; hence, although sampling was not designed to investigate virus distribution, this suggests that the presence of insect-only flaviviruses is patchy and potentially linked to a common source of infection. This would be in agreement with the hypothesis that insect-only flaviviruses can be transmitted vertically (Cook *et al.*, 2006). Indeed, evidence exists that West Nile virus can be transmitted vertically by *Culex* and *Stegomyia* mosquitoes (as *Aedes albopictus* and *Ae. aegypti*) (Baqar *et al.*, 1993). Although CxFV Uganda and NAKV were both isolated from female mosquitoes, these were collected by using CDC light traps supplemented with dry ice, therefore biasing sampling towards host-seeking females. Hence, the possibility that the viruses may also have been present in male mosquitoes, as described for CFAV in Puerto Rico (Cook *et al.*, 2006), cannot be discounted.

The development of a molecular ‘backbone’ phylogeny for the mosquitoes present in a given region of study, as conducted here, is essential for the screening of potential viral vectors, because many aedine and *Culex* species can only be identified by dissection of male genitalia. Without the development of a molecular system, particularly in species-rich tropical sampling areas, identification of entire adults via hand lens or microscope over a chill block in the field prior to viral screening may result in misidentification. This study has shown that primers LCO1490 and HCO2198 did not amplify the barcode region for *Ma. africana nigerrima*. Barcode primers have, to date, primarily been used for potential vectors of malaria, i.e. species of *Anopheles*. Future work will aim to tailor the system to all taxa of the family Culicidae.

Overall, considering that (i) many undiscovered insect-only flaviviruses are likely to exist in nature, (ii) flavivirus RNA has recently been discovered in phlebotomine sandflies (Moureau *et al.*, 2009) and (iii) the transmission dynamics of the insect-only flaviviruses remain unclear, but may involve vertical transmission, it is clear that this group requires further investigation. Whilst there is no evidence that they cause disease in humans, the high prevalence of the flaviviruses in general raises a number of issues for further consideration. First, future work should aim to investigate the effect of insect-only flaviviruses on different mosquito cell lines and on all life stages of vector species to clarify potential mechanisms of infection and vertical transmission. Second, the possibility of competitive exclusion amongst co-circulating flaviviruses within the vector population, including the effect of infection by insect-only flaviviruses on vector competence for other flaviviruses, also requires investigation via experimental-infection studies. Finally, given that insect-only flaviviruses appear to infect both mosquitoes (Culicidae) and phlebotomine sandflies (Psychodidae), it is advisable to screen potential vector species of both families from any given locality via an integrated approach.

## Supplementary Material

[Supplementary tables]

## Figures and Tables

**Fig. 3. f3:**
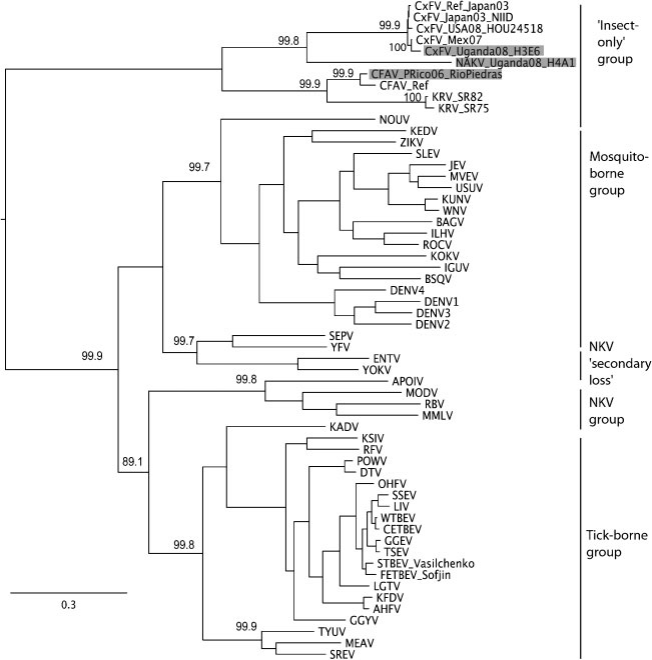
Bayesian phylogeny of the ORF amino acid dataset of the genus *Flavivirus*, including novel strain CxFV Uganda and NAKV. Posterior probabilities (%) are shown for the main clades only. See Supplementary Table S2 for virus abbreviations and GenBank accession numbers. All horizontal branch lengths are drawn to scale; bar, 0.3 substitutions per site. The tree is midpoint-rooted for purposes of clarity only. Those flavivirus sequences that are published for the first time in the current study, namely NAKV, CxFV strain Uganda and CFAV strain Rio Piedras, are highlighted in grey.

**Table 1. t1:** Results of investigation of DNA forms in NAKV and CxFV C6/36 cell culture

**Sample**	**Primers**	**No RT classic PCR**		**RT-PCR one-step**		**No RT classic PCR**	
		**Supernatant**	**Cells**	**Supernatant**	**Cells**	**Cells +DNase**	**Cells no DNase**
H4A1	NS3 verif S/R	−	+	+	+	−	+
	NS5 verifB S/R	−	+	+	+	−	+
H3E6	ENV verif S1/R1	−	+	+	+	−	+
	NS3 verif S/R	−	+	+	+	−	+
	NS5 verifB S/R	−	+	+	+	−	+
