# Supplementary material for: Isolation of a novel species of flavivirus and a new strain of Culex flavivirus (Flaviviridae) from a natural mosquito population in Uganda
Source: J Gen Virol. 2009 Nov;90(Pt 11):2669–78. doi: 10.1099/vir.0.014183-0 (PMC2885038; doi:10.1099/vir.0.014183-0)
Supplement: [Supplementary tables] [file supp_90_11_2669__index.html]

 Isolation of a novel species of flavivirus and a new strain of Culex flavivirus (Flaviviridae) from a natural mosquito population in Uganda -- Cook et al. 90 (11): 2669 Data Supplement - Supplementary tables -- Journal of General Virology

### Isolation of a novel species of flavivirus and a new strain of Culex flavivirus (*Flaviviridae*) from a natural mosquito population in Uganda, by S. Cook, G. Moureau, R. E. Harbach, L. Mukwaya, K. Goodger, F. Ssenfuka, E. Gould, E. C. Holmes and X. de Lamballerie

*Journal of General Virology* vol. **90**, part 11, pp. 2669–2678

  

**Supplementary Table S1.** Degenerate primer sequences used for viral screening and primer sets used for further investigation of DNA forms.

**Supplementary Table S2.** Flaviviral abbreviation codes and GenBank accession numbers.

**Supplementary Table S3.** Mosquito accession numbers and Barcode of Life Datasystem (BOLD) reference numbers.

[Single PDF file] (128 KB)

---

|  |  |  |
| --- | --- | --- |
| INT J SYST EVOL MICROBIOL | MICROBIOLOGY | J GEN VIROL |
| J MED MICROBIOL | ALL SGM JOURNALS | |
