# Supplementary material for: Isolation of a novel species of flavivirus and a new strain of Culex flavivirus (Flaviviridae) from a natural mosquito population in Uganda
Source: J Gen Virol. 2009 Nov;90(Pt 11):2669–78. doi: 10.1099/vir.0.014183-0 (PMC2885038; doi:10.1099/vir.0.014183-0)
Supplement: [Supplementary tables] [file supp_90_11_2669__1.pdf]

Supplementary Table S1. Degenerate primer sequences used for viral screening and primer sets used for further investigation of DNA forms

Primer	Gene	Sequence, 5′–3′	Polarity
PF1S	NS5	TGYRTBTAYAACATGATGGG	Sense
PF2R-bis	NS5	GTGTCCCAICCNGCNGTRTC	Reverse
PF3S	NS5	ATHTGGTWYATGTGGYTDGG	Sense
H4A1 NS3 verif S	NS3	TTGCCTGTCGAGGGATAATG	Sense
H4A1 NS3 verif R	NS3	GTCCGGGTGTCAATGACAGT	Reverse
H4A1 NS5 verifB S	NS5	ACGTACCATATGGTACATGTG	Sense
H4A1 NS5 verifB R	NS5	GATGCGAGACCTTCCTATTAT	Reverse
H3E6 ENV verif S1	ENV	CATCGTGATGAACATCACAGC	Sense
H3E6 ENV verif R1	ENV	GGAGAGGCACTAGTTATCAC	Reverse
H3E6 NS3 verif S	NS3	GGAAGTATCACTCCTTAATCAC	Sense
H3E6 NS3 verif R	NS3	GATGCCAACTTTCTCCAGCAT	Reverse
H3E6 NS5 verifB S	NS5	CGGATTATCTGGTACATGTGG	Sense
H3E6 NS5 verifB R	NS5	ATCTCGTGTTGGTCACGACA	Reverse

Supplementary Table S2. Flaviviral abbreviation codes and GenBank accession numbers

Flavivirus	Abbreviation	GenBank acccession no.		
		ORF	NS5	E
Alkhurma virus	AHFV	NC_004355	NC_004355	NC_004355
Apoi virus	APOIV	NC_003676	NC_003676	NC_003676
Bagaza virus	BAGV	AY632545	AY632545	AY632545
Banzi virus	BANV		L40951	
Bussuquara virus	BSQV	NC_009026	NC_009026	NC_009026
Cell fusing agent virus – reference strain	CFAV	NC_001564	NC_001564	NC_001564
Cell fusing agent virus strain Rio Piedras	CFAV	GQ165810	GQ165810	GQ165810
Culex flavivirus	CxFV			
Reference strain		NC_008604	NC_008604	NC_008604
Strain HOU242518		FJ502995	FJ502995	FJ502995
Strain HOU242519				FJ502996
Strain HOU24284				FJ502997
Strain HOU24471				FJ502998
Strain HOU24516				FJ502999
Strain HOU24522				FJ503000
Strain HOU24559				FJ503001
Strain Izabal			EU805806	EU805805
Strain Mex07		EU879060	EU879060	EU879060
Strain NIID-21-2		AB377213	AB377213	AB377213
Strain TR3115				FJ503002
Strain TR3116				FJ503003
Strain Uganda08		GQ165808	GQ165808	GQ165808

Cook, S., Moureau, G., Harbach, R. E., Mukwaya, L., Goodger, K., Ssenfuka, F., Gould, E., Holmes, E. C. & de Lamballerie, X. (2009). Isolation of a novel species of flavivirus and a novel strain of Culex flavivirus (*Flaviviridae*) from a natural mosquito population in Uganda. *J Gen Virol* **90**, 0000–0000.

Flavivirus	Abbreviation	GenBank acccession no.		
		ORF	NS5	E
Deer tick virus	DTV	AF311056	AF311056	AF311056
Dengue virus 1	DENV1	M87512	M87512	M87512
Dengue virus 2	DENV2	M19197	M19197	M19197
Dengue virus 3	DENV3	DQ675533	DQ675533	DQ675533
Dengue virus 4	DENV4	AY618993	AY618993	AY618993
Entebbe bat virus	ENTV	NC_008718	NC_008718	NC_008718
Gadgets gully virus	GGYV	DQ235145	DQ235145	DQ235145
Greek goat encephalitis virus	GGEV	DQ235153	DQ235153	DQ235153
Iguape virus	IGUV	NC_009027	NC_009027	NC_009027
Ilheus virus	ILHV	NC_009028	NC_009028	NC_009028
Japanese encephalitis virus	JEV	NC_001437	NC_001437	NC_001437
Kadam virus	KADV	DQ235146	DQ235146	DQ235146
Kamiti River virus strain KRV-75	KRV	AY149904	AY149904	AY149904
Kamiti River virus strain KRV-82	KRV	NC_005064	EU074051	NC_005064
Karshi virus	KSIV	NC_006947	NC_006947	NC_006947
Kedougou virus	KEDV	AY632540	AY632540	AY632540
Kokobera virus	KOKV	NC_009029	NC_009029	NC_009029
Kunjin virus	KUNV	AY274505	AY274505	AY274505
Kyanasur Forest disease virus	KFDV	AY323490	AY323490	AY323490
Langat virus	LGTV	NC_003690	NC_003690	NC_003690
Louping Ill virus	LIV	NC_001809	NC_001809	NC_001809
Meaban virus	MEAV	DQ235144	DQ235144	DQ235144
Modoc virus	MODV	NC_003635	NC_003635	NC_003635
Montana myotis leukoencephalitis virus	MMLV	NC_004119	NC_004119	NC_004119
Murray Valley encephalitis virus	MVEV	NC_000943	NC_000943	NC_000943
Nakiwogo virus strain UG134-26	NAKV	GQ165809	GQ165809	GQ165809
Nounane virus	NOUV	EU159426	EU159426	EU159426
Omsk hemorrhagic fever virus	OHFV	NC_005062	NC_005062	NC_005062
Powassan virus	POWV	NC_003687	NC_003687	NC_003687
Rio Bravo virus	RBV	NC_003675	NC_003675	NC_003675
Rocio virus	ROCV	AY632542	AY632542	AY632542
Royal Farm virus	RFV	DQ235149	DQ235149	DQ235149
Saumarez Reef virus	SREV	DQ235150	DQ235150	DQ235150
Sepik virus	SEPV	NC_008719	NC_008719	NC_008719
Spanish sheep encephalitis virus	SSEV	DQ235152	DQ235152	DQ235152
St Louis encephalitis virus	SLEV	NC_007580	NC_007580	NC_007580
Tick-borne encephalitis virus	TBEV	NC_001672	NC_001672	NC_001672
Tick-borne encephalitis virus Hypr	CETBEV_Hypr	U39292	U39292	U39292
Tick-borne encephalitis virus strain Vasilchenko	STBEV_Vasilchenko	AF069066	AF069066	AF069066
Tick-borne encephalitis virus strain Sofjin	FETBEV_Sofjin	AB062064	AB062064	AB062064
Turkish sheep encephalitis virus	TSEV	DQ235151	DQ235151	DQ235151
Tyuleniy virus	TYUV	DQ235148	DQ235148	DQ235148
Usutu virus	USUV	NC_006551	NC_006551	NC_006551
West Nile virus	WNV	NC_001563	NC_001563	NC_001563
Western Tick-borne encephalitis virus	WTBEV	NC_001672	NC_001672	NC_001672
Yellow Fever virus	YFV	NC_002031	NC_002031	NC_002031
Yokose virus	YOKV	NC_005039	NC_005039	NC_005039
Zika virus	ZIKV	EU545988	EU545988	EU545988

Cook, S., Moureau, G., Harbach, R. E., Mukwaya, L., Goodger, K., Ssenfuka, F., Gould, E., Holmes, E. C. & de Lamballerie, X. (2009). Isolation of a novel species of flavivirus and a novel strain of Culex flavivirus (*Flaviviridae*) from a natural mosquito population in Uganda. *J Gen Virol* **90**, 0000–0000.

Supplementary Table S3. Mosquito accession numbers and Barcode of Life Datasystem (BOLD) reference numbers for species shown in Fig. 6

Voucher samples collected in Uganda are identified by specimen numbers beginning with ‘V’.

Species/strain	GenBank accession no.
**Fly region**	
Anopheles braziliensis	DQ913835
Anopheles darlingi	DQ076235
Anopheles funestus	DQ287358
Anopheles minimus	AF417710
Anopheles nuneztovari	AF270929
Anopheles quadrimaculatus	NC_000875
Armigeres subalbatus	AY440299
Chagasia bathana	AF417726
Culex decens	AY645241
Ochlerotatus atropalpus	AF425845
Sabethes cyaneus	AF425840
Toxorhynchites amboinensis	AF417727
Toxorhynchites sp.	AF425850
Uranotaenia iowii	AF417728
Culex quinquefasciatus V215	GQ165759
Culex quinquefasciatus V216	GQ165760
Culex quinquefasciatus V217	GQ165761
Culex quinquefasciatus V219	GQ165762
Culex quinquefasciatus V221	GQ165763
Culex quinquefasciatus V222	GQ165764
Culex quinquefasciatus V223	GQ165765
Culex quinquefasciatus V224	GQ165766
Coquilletidia maculipennis V12	GQ165767
Anopheles implexus V153	GQ165768
Coquilletidia metallica V17	GQ165769
Eretmapodites intermedius V197	GQ165770
Mansonia africana H4A1	GQ165771
Mansonia africana H4D1	GQ165772
Mansonia africana V227	GQ165773
Mansonia uniformis V226	GQ165774
Coquilletidia fuscopennata V236	GQ165775
Coquilletidia aurites V237	GQ165776
Coquilletidia aurea V2	GQ165777
Culex cinereus V46	GQ165778
Eretmapodites intermedius V48	GQ165779
Culex annulioris V69	GQ165780
Stegomyia denderensis V79	GQ165781
Stegomyia africana V80	GQ165782
Stegomyia aegypti V95	GQ165783
Culex quinquefasciatus H3E6	GQ165784

Cook, S., Moureau, G., Harbach, R. E., Mukwaya, L., Goodger, K., Ssenfuka, F., Gould, E., Holmes, E. C. & de Lamballerie, X. (2009). Isolation of a novel species of flavivirus and a novel strain of Culex flavivirus (*Flaviviridae*) from a natural mosquito population in Uganda. *J Gen Virol* **90**, 0000–0000.

Species/strain	GenBank accession no.
**Barcode region**	
Culiseta minnesotae	ACMC226-04| mosqMN55mF4|
Culiseta morsitans	ACMC071-04|mosq184-70|
Culiseta impatiens	AF425848
Culex malayi	GBDP2247-06|DQ149238|
Culex minor	GBDP1888-06|AY917211|
Culex rubithoracis	GBDP1680-06|AY729981|
Culex territans	ACMC283-04|mosqTT41
Culex brevipalpis	GBDP1810-06|AY834238
Culex minutissimus	GBDP2249-06|DQ149240|
Culex infantulus	GBDP1665-06|AY729966|
Culex annulioris V69	GQ165805
Culex fuscocephala	GBDP2245-06|DQ149236|
Culex infula	GBDP1682-06|AY729983|
Culex bitaeniorhynchus	GBDP2368-06|DQ267687|
Culex murrelli	GBDP1667-06|AY729968|
Culex palpalis	GBDP3004-07|DQ673813|
Culex annulirostris	GBDP3132-07|DQ673685|
Culex gelidus	GBDP1664-06|AY729965|
Culex sitiens	GBDP2964-07|DQ673853|
Culex cornutus	GBDP1815-06|AY834243|
Culex cinereus V43	GQ165804
Culex nigropunctatus	GBDP1675-06|AY729976|
Culex pallidothorax	GBDP2252-06|DQ154154|
Culex vishnui	GBDP1891-06|AY917214|
Culex pseudovishnui	GBDP1820-06|AY834248|
Culex tritaeniorhynchus	GBDP1883-06|AY917206|
Culex whitmorei	GBDP2265-06|DQ154167|
Culex fuscanus	GBDP1684-06|AY729985|
Culex tarsalis	AF425847
Culex salinarius	ACMC268-04|mosqSN37|
Culex modestus	GBDP4838-08|FM177758|
Culex hutchinsoni	GBDP2248-06|DQ149239|
Culex torrentium	AM403477
Culex pipiens 1	DQ360492
Culex pipiens 2	ACMC232-04|mosqPP4|
Culex pipiens 3	DQ360492
Culex pipiens 4	ACMC234-04|mosqPP6|
Culex pipiens 5	ACMC235-04|mosqPP7|
Culex pipiens 6	GBDP4839-08|FM177757
Culex pipiens 7	AM403492
Culex pipiens 8	FM177757
Culex pipiens 9	AM403476
Culex quinquefasciatus 1	GBDP4840-08|FM177756|
Culex quinquefasciatus 2	GBDP1676-06|AY729977|
Culex quinquefasciatus 3	FM177756
Culex quinquefasciatus 4	GBDP2370-06|DQ267689|
Culex quinquefasciatus 5	AY729977

Cook, S., Moureau, G., Harbach, R. E., Mukwaya, L., Goodger, K., Ssenfuka, F., Gould, E., Holmes, E. C. & de Lamballerie, X. (2009). Isolation of a novel species of flavivirus and a novel strain of Culex flavivirus (*Flaviviridae*) from a natural mosquito population in Uganda. *J Gen Virol* **90**, 0000–0000.

Species/strain	GenBank accession no.
Culex quinquefasciatus V215	GQ165791
Culex quinquefasciatus V216	GQ165792
Culex quinquefasciatus V217	GQ165793
Culex quinquefasciatus V219	GQ165794
Culex quinquefasciatus V220	GQ165795
Culex quinquefasciatus V222	GQ165796
Culex quinquefasciatus V223	GQ165797
Culex quinquefasciatus V224	GQ165798

Cook, S., Moureau, G., Harbach, R. E., Mukwaya, L., Goodger, K., Ssenfuka, F., Gould, E., Holmes, E. C. & de Lamballerie, X. (2009). Isolation of a novel species of flavivirus and a novel strain of Culex flavivirus (*Flaviviridae*) from a natural mosquito population in Uganda. *J Gen Virol* **90**, 0000–0000.
